# Emotional Lability at Disease Onset Is an Independent Prognostic Factor of Faster Disease Progression in Amyotrophic Lateral Sclerosis

**DOI:** 10.14336/AD.2019.1120

**Published:** 2020-10-01

**Authors:** Krzysztof Barć, Katarzyna Szacka, Krzysztof Nieporęcki, Mamede de Carvalho, Marta Gromicho, Julian Grosskreutz, Susanne Petri, Annekathrin Rödiger, Robert Steinbach, Hilmi Uysal, Magdalena Kuźma-Kozakiewicz

**Affiliations:** ^1^Department of Neurology, University Clinical Centre of Medical University of Warsaw, Warsaw, Poland; ^2^Department of Neurology, Medical University of Warsaw, Warsaw, Poland; ^3^Faculdade de Medicina-IMM, Universidade de Lisboa, Lisbon, Portugal; ^4^Hans-Berger Department of Neurology, Jena University Hospital, Germany; ^5^Department of Neurology, Hannover Medical School, Hannover, Germany; ^6^Department of Neurology, Akdeniz University Faculty of Medicine, Antalya, Turkey; ^7^Neurodegenerative Diseases Research Group, Medical University of Warsaw, Warsaw, Poland

**Keywords:** amyotrophic lateral sclerosis, emotional lability, pseudobulbar affect, pathological laughing and crying, disease progression, prognosis, epidemiology

## Abstract

Amyotrophic lateral sclerosis (ALS) is a fast progressing neurodegenerative disease leading to quadriplegia, anarthria and respiratory insufficiency. A large variety of phenotypes and disability progression requires individually tailored management. Identification of predictors of poor prognosis may not only improve management, but also allow for more precise patients’ stratification for clinical trials or research studies. The aim of the study was to investigate the influence of emotional lability present at disease onset on ALS progression by exploring its direct impact on the decay of the ALS Functional Rating Scale-Revised (ALSFRS-R). The study was performed in a group of 1145 patients from Germany, Poland, Portugal and Turkey between 2014 and 2018. The analysis showed that the presence of emotional lability at ALS onset was linked to a faster decline of ALSFRS-R (0.70 vs 0.50, p<0.0001), in case of either bulbar (0.80 vs 0.65, p<0.05) or limb disease onset (0.59 vs 0.46, p <0.01). It was most prominent in the bulbar subscore of ALSFRS-R. A multiple regression analysis showed a direct influence of emotional lability at ALS onset on disease progression, regardless of age, gender, site of onset, weight loss, cognitive impairment and diagnosis delay (β=0.071; p=0.019). It can therefore be concluded that the presence of emotional lability at the disease onset is an independent factor of faster disease progression in ALS.

Amyotrophic lateral sclerosis (ALS) is a fast progressing neurodegenerative disease leading to quadriplegia, anarthria and respiratory insufficiency. A large variety of phenotypes and progression of disability requires individually tailored management. Numerous studies are conducted every year with the scope to understand the disease pathogenesis and identify the most promising pathways and molecular targets [1-3]. Clinical significance of these studies is determined by accurate patients’ characterisation, taking into account not only the demographic and clinical factors, but also predictors of a poorer prognosis, in order to exclude their direct influence on the study outcome [4-6].

Beside motor neurons, ALS may involve the frontotemporal cortex, as well as extrapyramidal and autonomous systems [7, 8]. The extra-motor involvement in ALS is mainly subclinical [9]. The main non-motors symptoms of ALS include cognitive impairment (language and executive dysfunction) and emotional lability (EL), also known as pseudobulbar affect, pathological laughing and crying or emotional incontinence [10]. EL may be either present at ALS onset or develop with the disease progression. It occurs in up to 50% of patients throughout the disease course and is more frequently present in females [11]. The mechanism of EL remains unclear, as it probably involves damage of motor and temporal cortex and the disinhibition of brainstem and a putative centre for laughing and crying [11-15]. EL is characterised by involuntary outbursts of crying and/or laughing (less commonly). The episodes usually appear spontaneously or are triggered by minor emotional stimuli and cannot be controlled [10]. Since patients preserve insight into their condition, the emotional outbursts cause major distress, affecting the quality of the everyday-life. The treatment of EL comprises antidepressive drugs, including tricyclic antidepressants (amitriptyline, nortriptyline) and selective serotonin reuptake inhibitors (fluoxetine, sertraline, citalopram, paroxetine). A combination of dextromethorphan and quinidine, affecting glutamate and serotonin pathways, has been shown effective in reducing the laughing/crying episodes as well as improving the quality of life and relationships in ALS patients [16, 17]. The treatment is only partially effective.

In the majority of cases, EL occurs independently of depression, however the two conditions may coexist in some patients [18]. As different treatment approaches may be required, it is important to distinguish the EL from mood alterations. The clinical evaluation can be aided by diagnostic tools. It has been shown that the Center for Neurologic Study-Lability Scale (CNS-LS) and Hamilton Rating Scale for Depression (HRSD) do not correlate in ALS patients, which suggests a different pathomechanism of EL and affective disorders [18]. As up to 50% of ALS patients may experience cognitive impairment, the early detection of behavioral disturbance may help introduce the optimal care. Importantly, at early stages the dementia with a predominant involvement of the prefrontal cortex may mimic the episodes of EL, as frequent mood changes may be present in both conditions. The differential diagnosis may require a detailed assessment of a patient’s functional and cognitive capabilities. It needs to be emphasized that EL is considered a separate disorder, as no correlation has been found in any aspect of cognitive profile and EL in ALS patients [19].

The aim of our study was to assess the impact of EL at ALS onset on disease progression. To our knowledge, this is the first study concerning the impact of EL on the disease progression rate.

## MATERIALS ANS METHODS

### Patients

The analysis included 1145 consecutive patients from Germany, Poland, Portugal and Turkey with definite, probable or possible ALS according to the revised El Escorial criteria [20]. The data were collected cross-sectionally over a period of 4 years within the JPND project ONTology-based Web Database for Understanding Amyotrophic Lateral Sclerosis - ONWebDUALS. The disease onset was defined as the occurrence of the first muscles weakness. The disease duration was measured from the time of the first symptoms onset till the date of the clinical evaluation. EL at disease onset (ELO) was assessed based on a direct question whether the patient had experienced outbursts of uncontrollable laughing and/or crying from the beginning of the disease, accompanied by a detailed description of EL as the occurrence of laughing and/or crying episodes due to adequate although mild stimuli (p.ex. talking about the family/disease, watching movies/sports) or even without thinking of anything happy or sad, with a feeling of lability in controlling the presentation of emotions, without a concurrent alteration in the experience of emotions (mood alterations). In the vast majority cases the obtained information was confirmed by or obtained in the presence of the caregiver/s. The weight loss was defined as a reduction of the body weight of at least 10% in 36 months before the onset of the symptoms. The depression and cognitive impairment were assessed as binary variables (presence or absence of depression or cognitive impairment, respectively). It followed an individual discussion with the patient. For “depression” it concerned constant mood disturbances, feeling of worthlessness or excessive guilt, loss/increase of appetite, night rest/sleep alterations, loss of energy or interest, with each symptom experienced for a period of at least 2 weeks. For the “cognitive impairment” it included the presence of memory deficits, language impairment, personality or behavioural changes, impaired ability to perform daily tasks.

In order to confirm the significance of the above evaluations, in a subgroup of patients the results were compared to the Edinburgh Cognitive and Behavioural ALS Screen (ECAS, n=191) and the ALS Depression Inventory (ADI-12, n=62). We also examined the differences between patients with and without EL with regard to ECAS and ADI-12 scales.

**Table 1:** Patients’ baseline characteristics.

	All patients (n=1145)	ELO(+) group (n=199)	ELO(-) group (n=946)	p-value
Demographic characteristics *Gender (%)* Female Male	44.28 55.72	51.76 48.24	42.71 57.29	<0.05*
*Age at time of evaluation (years ± SD)*	61.6±12.79	61.7±12.32	61.6±12.89	=0.93*
Disease characteristics *Site of onset (%)* Bulbar Limb	27.25 72.75	45.73 54.27	23.36 76.64	<0.0001*
*Emotional lability at onset (%)* Yes No	17.38 82.62	100 -	- 100	-
*Cognitive impairment at onset (%)* Yes No	11.70 88.30	32.16 67.84	7.40 92.60	<0.0001*
*Initial weight loss (%)* Yes No	17.47 82.53	29.15 70.85	15.00 85.00	<0.0001*

* Chi-square test (for categorical variables) and t-Student (for continues variables). ELO(+) = patients with emotional lability at disease onset; ELO(-) = patients without emotional lability at disease onset.

### Disease progression

The disease progression was evaluated with ALSFRS-R (ALS Functional Rating Scale Revised) decline rate, calculated as a quotient of a difference between ALSFRS-R score at symptoms onset (presumed as normal score of 48 points) and ALSFRS-R score at time of patient’s evaluation by a difference between the date of evaluation and the date of symptoms onset expressed in months [48 - ALSFRS-R/the date of clinical evaluation - the date of disease onset]. The diagnosis delay was defined as the time from the first symptoms onset to the ALS diagnosis.

### Statistical analysis

Chi-square and t-Student tests were used for the comparisons between groups. Binary predictors of faster progression were investigated with Mann-Whitney U-test and continuous predictors with simple regression analysis. A two-way ANOVA was conducted to detect a interaction between depression and EL concerning disease progression. Multiple regression analysis was applied to explore the direct influence of all confounding factors. A p-value of 0.05 was considered for statistical significance. Analyses were performed in SPSS v25.0 software (IBM SPSS Statistics).

**Table 2:** ALSFRS-R decline rate in terms of bulbar, motor, and respiratory function subscore.

	ALSFRS-R bulbar subscore decline rate (median)		p-value*	ALSFRS-R motor subscore decline rate (median)		p-value*	ALSFRS-R respiratory subscore decline rate (median)		p-value*
	ELO(+)	ELO(-)		ELO(+)	ELO(-)		ELO(+)	ELO(-)	
All patients	0.21	0.06	<0.0001	0.39	0.34	=0.14	0.03	0.00	<0.005
Bulbar onset patients	0.39	0.31	<0.05	0.33	0.23	=0.16	0.08	0.00	<0.05
Limb onset patients	0.07	0.01	<0.0001	0.43	0.36	=0.06	0.00	0.00	=0.41

* Mann-Whitney test. ALSFRS-R = Amyotrophic Lateral Sclerosis Functional Rating Scale - Revised; ELO(+) = patients with emotional lability at disease onset; ELO(-) = patients without emotional lability at disease onset.

## RESULTS

### Baseline characteristics

The mean age at the time of evaluation was 61.6 (SD ± 12.79) years, with male to female ratio of 1.26. Bulbar onset was reported in 27.25% of patients and cognitive impairment at disease onset in 11.70% cases (Table 1). The median diagnosis delay was 11.01 months (Q1-Q3, 5.98 - 20.96).

ELO was present in 17.38% patients [ELO(+) group]. There were significantly more females, individuals with bulbar onset, initial weight loss and cognitive impairment as compared to ALS patients without ELO [ELO(-) group]. The groups did not differ with regard to age (Table 1).

**Table 3:** The comparison of ALSFRS-R decline rates between patients with and without emotional lability at disease onset in terms of disease onset, weight loss and gender.

	ALSFRS-R decline rate (median)		p-value*
	ELO(+) (n=199)	ELO(-) (n=946)	
All patients	0.70	0.50	<0.0001
Bulbar onset Limb onset	0.80 0.59	0.65 0.46	<0.05 <0.01
Weight loss at onset Stable weight at onset	0.83 0.64	0.65 0.48	<0.05 <0.001
Female Male	0.78 0.61	0.56 0.48	<0.001 <0.05

* Mann-Whitney test. ALSFRS-R = Amyotrophic Lateral Sclerosis Functional Rating Scale - Revised; ELO(+) = patients with emotional lability at disease onset; ELO(-) = patients without emotional lability at disease onset.

### Subgroup evaluation of the presence of cognitive impairment and depression

There was no significant difference between the subgroup assessed with ECAS (n=191) and our cohort (n=1145) with regard to age (p=0.59), gender (p=0.87), type of onset (p=0.87), occurrence of initial weight loss (p=0.08), diagnostic delay (p=0.85) and the decay of ALSFRS-R (p=0.18). The subgroup evaluated with ADI-12 scale (n=62) did not differ relevantly from our cohort (n=1145) in terms of gender (p=0.66), type of onset (p=0.43), occurrence of initial weight loss (p=0.53), diagnostic delay (p=0.49) and the decay of ALSFRS-R (p=0.48), but it was characterised by younger age (p=0.04).

The mean score of ECAS was significantly lower in the subgroup of patients evaluated as “cognitively impaired” based on the questionnaire, as compared to “cognitively normal” both at the time of evaluation (68.4 [SD±19.31] *vs* 95.8 [SD±23.56], p<0.0001) and at the disease onset (82.3 [SD±31.18] vs 93.8 [SD±22.34], p=0.03). Significantly higher mean ADI-12 score was found in the group qualified with “the presence of depression” based on the questionnaire as compared to the group assessed with “the absence of depression” (27.3 [SD±5.52] *vs* 20.3 [SD±5.44], p<0.0001). As this subgroup differed in terms of age from our cohort, we adjusted this data for age, with no observed influence on the obtained result.

### Cognitive impairment, depression and emotional lability

No significant differences of the ECAS mean score were found between the EL(+) and EL(-) group either at the disease onset (92.7 [SD±22.79] vs 92.3 [SD±24.13], p=0.96) nor at the time of evaluation (91.3 [SD±26.86] vs 92.7 [SD±23.30], p=0.76). In addition, no relevant difference was found between the mean ADI-12 score obtained in the EL(+) and EL(-) group at the time of evaluation (25.0 [SD±8.74] *vs*25.7 [SD±4.78], p=0.67).

### Emotional lability as the predictor of ALS prognosis

The ELO(+) was linked to a higher ALSFRS-R decline rate in the course of the disease as compared to ELO(-) patients (0.70 vs 0.50, p<0.0001). The decay of ALSFRS-R was faster in all three subscores: bulbar, motor (without reaching statistical significance) and respiratory. In particular, bulbar onset ALS patients had faster decline of bulbar (0.39 vs 0.31, p<0.05) and respiratory subscores of ALSFRS-R (0.08 vs 0.00, p<0.05), while limb onset patients presented faster decay of bulbar (0.07 vs 0.01, p<0.0001) subscore of ALSFRS-R (Table 2). A subsequent analysis of each variant group of binary predictors, including disease onset, initial weight loss and gender, revealed a significantly higher ALSFRS-R decline rate in ELO(+) patients (Table 3).

**Table 4:** Binary predictors of faster progression of the disease.

Predictor	ALSFRS-R decline rate (median)	n	p-value*
Disease onset Bulbar Limb	0.70 0.48	312 833	<0.0001
Gender Female Male	0.62 0.49	507 638	<0.0001
Initial weight loss^1^ Yes No	0.71 0.47	200 945	<0.0001
Cognition impairment at onset Yes No	0.58 0.54	134 1011	=0.30

* Mann-Whitney test. ^1^>10% in 36 months before disease onset. ALSFRS-R = Amyotrophic Lateral Sclerosis Functional Rating Scale - Revised.

### Other predictors of faster progression

We found that bulbar onset (0.70 vs 0.48, p<0.0001), female gender (0.62 vs 0.49, p<0.0001) and initial weight loss (0.71 vs 0.47, p<0.0001) were binary predictors of faster progression in ALS (Table 4). Cognitive impairment at disease onset resulted in a slightly higher decay of ALSFRS-R but did not reach statistical significance. Age (p<0.001) and diagnosis delay (p<0.001) were both found to be continuous factors of faster disease progression.

**Table 5:** Multiple regression analysis for the predictors of faster ALSFRS-R decline rate.

Predictor	Unstandardized coefficients	Standardized coefficients	t-value	p-value
Emotional lability at onset	0.199	0.071	2.342	0.019
Gender	0.086	0.041	1.421	0.156
Site of onset	0.115	0.050	1.650	0.099
Initial weight loss	0.041	0.075	2.580	0.010
Diagnostic delay	0.010	0.260	9.008	<0.001
Age	0.009	0.108	3.686	<0.001
Cognitive impairment at onset	0.041	0.037	1.243	0.214

### Depression and emotional lability

We investigated a relationship between the presence of EL and depression at the time of patients’ evaluation in regard to disease progression. A two-way ANOVA showed an independent impact of EL (p=0.007) and depression (p=0.006) on ALSFRS-R decline rate, with no interaction between the two factors (p=0.541).

### Multiple regression analysis

Since predictors of faster ALSFRS-R decline rate were found more commonly in ELO(+) group, we investigated a direct influence of all confounding factors on the disease progression. A multiple regression analysis, adjusted for age, gender, site of onset, diagnosis delay, initial weight loss, ELO and cognitive impairment at disease onset, was performed showing a significant association of ELO(+) with further decay of ALFFRS-R (β=0.071, p=0.019) (Table 5).

## DISCUSSION

Due to its clinical characteristics, EL causes important distress to ALS patients and their proxy. For this reason, the majority of publications focus on the psychological, social and therapeutic aspects of the syndrome [10, 11, 15, 19, 21, 22]. Although the EL does not correlate with depression [18, 23-25], an ascending social isolation may indeed result in development of mood alterations, depression and further functional impairment [26, 27]. The EL at disease onset was present in 17.38% of individuals what constitutes over one third of ALS patients ever developing EL [11, 46-50].

For the first time we have shown that EL at the disease onset is an independent predictor of faster disease progression in ALS. In particular, the presence of ELO in patients with bulbar onset resulted mainly in a more pronounced progression of bulbar and respiratory dysfunction, while in patients with limb onset - the bulbar muscles involvement. The risk of developing EL at the disease onset was significantly higher for patients with bulbar onset ALS. As in numerous studies the bulbar onset had been linked to a faster progression of ALS, especially at the early disease stage [28, 29], the faster disease progression in ELO(+) may result from a predominant damage of the brainstem in these patients. However, we have observed a significantly faster decay of ALSFRS-R in the bulbar onset subgroup with ELO(+) as compared to bulbar onset ELO(-) patients. That concludes that the EL is a site-of-onset independent factor of faster progression in ALS. Moreover, our observation supports the hypothesis of a distinct mechanism involved in the development of EL and the bulbar palsy due to the damage in the brainstem. The dysregulation of emotional expression without the disturbance of emotional experience is the principal feature of EL. Various anatomical structures have been reported to be involved in the pathomechanism of EL, including the prefrontal cortex, the anterior cingulate, the internal capsule, the thalamus, the subthalamic nucleus the cerebellum and, most importantly, the paramedial part of the brainstem [30]. The pathomechanism is complex, as either a lesion or a stimulation of any of these structures may result in a sudden impairment of emotional expression without concurrent change of the individual’s feelings [31-37]. Finally, it is unclear why some patients develop solely either laughter or crying episodes, while others experience both forms of emotional incontinence [11].

Although we have not collected data regarding the occurrence of depression at the disease onset, we have assessed a relationship between the depression and the presence of EL at the time of patients’ evaluation. Both factors were found to independently affect ALSFRS-R decline rate in two-way ANOVA analysis.

To date, many demographic and disease characteristics as predictors of poorer prognosis in ALS have been established, including older age, female gender, unmarried status, initial weight loss (or overweight), diagnostic delay, depression and reduced slow vital capacity [26, 38-43]. While some of them are commonly reported (e.g. bulbar onset, initial weight loss), others remain inconclusive (e.g. female gender, depression). In our cohort, we have observed the influence of older age, female gender, bulbar onset, initial weight loss and shorter diagnostic delay on the disease progression. As opposed to other studies [28, 29], bulbar onset did not reach statistical significance (p=0.099) in applied multiple regression model, as the older age and shorter diagnostic delay were more frequently present in bulbar onset group.

The main limitation of the current study is the lack of linearity of ALSFRS-R decay in the disease course and the inhomogeneity of disease progression among individual ALS patients [44]. It is not certain if prognostic factors affecting ALSFRS-R decline rate influence the survival. To date, there has been only one study describing a possible impact of EL on the ALS outcome [45]. It showed a slightly shorter survival of patients with EL, without reaching statistical significance (p=0.25). The study did not involve the analysis of the disease progression. Its major limitation was a small sample size (n=94). Larger group studies are needed to explore the relationship between EL and the survival. Secondly, although the assessment of the EL was preceded by a thorough explanation of this phenomenon to individual patients, the question itself may not be fully EL-specific, as alike episodes may be observed in patients with cognitive impairment or chronic mood disturbance. Although there was a high frequency of EL among patients with cognitive impairment in our cohort, we found comparable results of ECAS among patient with and without EL. It is in agreement with the data previously reported by Palmieri et al. 2000 [19], who showed no correlation between EL and cognitive profile. We further found no differences in the ADI-12 scores in terms of the occurrence of EL. This all contributes to qualifying EL as a distinct condition from the cognitive impairment and mood disturbance disorders.

It remains unclear how the EL contributes to the faster progression of the disease. It may depend on additional damage to neuronal cells, a greater involvement of mechanism particularly affecting emotional control areas, or a psychological influence on personal attitude towards the disease resulting in a poorer outcome [10, 15, 26].

In conclusion, the presence of EL at ALS onset results in faster disease progression regardless of other known demographic and clinical prognostic predictors. Thus, the presence of EL requires more insightful care of ALS patients, including monitoring of bulbar involvement and respiratory function. Moreover, our results support the hypothesis of the distinct pathomechanism involved in the development of EL in ALS patients.
